# Refractory Mycoplasma Pneumonia in Children: A Systematic Review and Meta-analysis of Laboratory Features and Predictors

**DOI:** 10.1155/2022/9227838

**Published:** 2022-06-26

**Authors:** Wanjie Huang, Xianhong Xu, Wenqi Zhao, Qi Cheng

**Affiliations:** Department of Pediatrics, Shengjing Hospital of China Medical University, Shenyang, Liaoning, China

## Abstract

**Background:**

Mycoplasma pneumoniae is a common pathogen of community-acquired pneumonia (CAP) in children. *M. pneumoniae* infection is usually regarded as a self-limiting disease, but in some special cases, it can also develop into refractory Mycoplasma pneumoniae pneumonia (RMPP). The aim of this study is to analyze the clinical characteristics of CRP (C-reactive protein), LDH (lactate dehydrogenase), ESR (erythrocyte sedimentation rate), *D-dimer*, neutrophils (%), lymphocytes (%), and lung consolidation in RMPP and explore their prediction results in the early stage of RMPP, which is important for early treatment.

**Methods:**

This systematic search was conducted in PubMed, Embase, Cochrane Library, Web of Science, CNKI, Wangfang, and Cqvip, and the date was set until February 23, 2021. For the continuous variables, mean difference (MD) with 95% CI was adopted to evaluate CRP, LDH, ESR, D-dimer, neutrophils (%), lymphocytes (%), and the correlation between lung consolidation and RMPP.

**Results:**

20 studies including 5289 patients were included in the analysis, and the results showed that the CRP of the RMPP group (MD (95% CI): 22.29 (12.20, 32.38), *P* < 0.001), LDH (MD (95% CI): 145.13 (78.62, 211.64), *P* < 0.001), neutrophils (%) (MD (95% CI): 7.27 (0.31, 14.23), *P* = 0.04), and D-dimer (MD (95% CI): 1.79 (-1.17, 4.74), *P* = 0.24) was higher than that of the NRMPP group; the risk of lung consolidation in the RMPP group (OR (95% CI): 14.29 (4.52, 45.12), *P* < 0.001) was higher than that in the NRMPP group, and there was no difference in ESR (MD (95% CI): 8.11 (-1.34, 17.56), *P* = 0.09) and lymphocytes (%) (MD (95% CI): -6.27 (-12.81, 0.27), *P* = 0.06) between the two groups.

**Conclusion:**

So, the available evidence indicates that CRP, LDH, neutrophils (%), *D-dimer*, and lung consolidation are predictive factors for RMPP.

## 1. Introduction

Mycoplasma pneumoniae (*M. pneumoniae*) is a common pathogen of community-acquired pneumonia (CAP) in children [1, 2]. Studies have found that 10% to 30% of community-acquired pneumonia in children is caused by mycoplasma pneumonia [1].

*M. pneumoniae* infection is usually regarded as a self-limiting disease, but in some special cases, it can also develop into refractory Mycoplasma pneumoniae pneumonia (RMPP). As a special classification of *M. pneumoniae* pneumonia, RMPP has attracted worldwide attention and has become a clinical research hotspot. The definition standard of RMPP is still not unified. At present, it is usually defined as children with *M. pneumoniae* pneumonia after 7 days of treatment with macrolide antibiotics and have aggravated clinical signs, continued fever, aggravated pulmonary imaging findings, and extrapulmonary complications [3]. After a series of treatments, patients with refractory *M. pneumoniae* pneumonia may still develop necrotizing pneumonia, atelectasis, etc., and can even lead to death in severe cases [4]. Therefore, early identification and diagnosis of RMPP is a major problem that clinicians need to solve urgently. Some studies have shown that CRP, LDH, ESR, neutrophils (%), and lymphocytes (%) are predictive factors for RMPP [5–7], but it is contradictory that some studies have found no difference for these factors in RMPP and NRMPP [8, 9]. In addition, we have also found that a large-scale dense consolidation shadow of the lungs occurs in the RMPP population, which may also be one of the predictive factors for RMPP [10].

Based on the available evidence, we conducted a systematic review to study some predictive factors of RMPP and a retrospective analysis of the accuracy of some biomarkers in the diagnosis of RMPP to help clinicians find and treat RMPP as soon as possible.

## 2. Methods

### 2.1. Patient and Public Involvement

Patients and the public were not involved in planning the design and conducting, reporting, or disseminating the results of our study.

### 2.2. Search Strategies

The databases we searched included PubMed, Embase, Cochrane Library, Web of Science, CNKI, Wangfang, and Cqvip, and the search time was set to February 23, 2021. The search strategy was “Mycoplasma pneumoniae pneumonia” OR “MPP” OR “refractory Mycoplasma pneumoniae pneumonia” OR “RMPP” AND “children” OR “child”. The specific search algorithm is provided in Supplemental.

### 2.3. Inclusion and Exclusion Criteria

The inclusion criteria included (1) case-control study, (2) children with RMPP in the case group and children with nonrefractory Mycoplasma pneumoniae pneumonia (NRMPP) in the control group, and (3) Chinese articles (core journal) and English articles. Exclusion criteria included (1) meta, review, case report, comment, meeting abstract, or letter; (2) valid data could not be extracted from the study; and (3) animal experimental research.

### 2.4. Data Extraction and Quality Assessment

The data were extracted by two researchers (Xu and Huang) according to the inclusion and exclusion criteria. If conflicting data was found, the third researcher was selected to make the extraction judgment. The extracted information included first author, year of publication, country, the total number of case group/control group, age, gender, and quality score. Two researchers used the modified Newcastle–Ottawa Scale (NOS) to score independently. The total NOS score was 9 points, and the studies with scores < 6 points and ≥ 6 points were considered as low, medium, and high quality, respectively.

### 2.5. Statistical Analysis

The meta-analysis was performed using the STATA 15.1 software and Review Manager version 5.4.0. The heterogeneity test was adopted to evaluate various indicators. When the heterogeneity statistic *I*^2^ < 50%, the fixed effects model was used; otherwise, the random effects model was used. Mean difference (MD) and 95% CI were used as effect indicators. Sensitivity analysis was performed on all models, and publication bias was tested by Begg's test and Egger's test. The difference was statistically significant with *P* < 0.05.

## 3. Results

### 3.1. Literature Search and Study Selection

**Figure 1:**
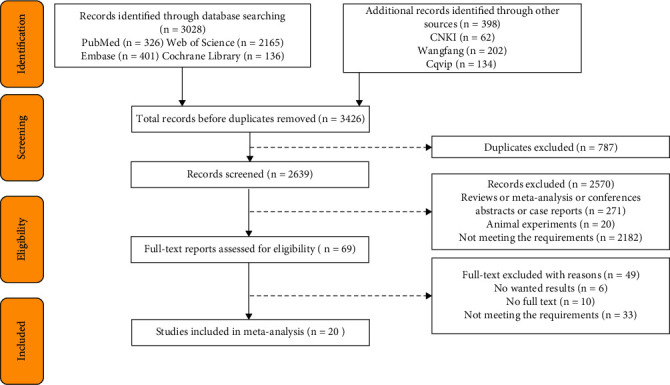
Retrieval flow chart.

Through database search, 3426 articles in total were retrieved. After the deletion of duplicate articles, 2639 articles were left, and 20 case-control studies were selected according to the inclusion and exclusion criteria [3, 10–23].  Figure 1 shows the detailed retrieval process.

A total of 5289 patients participated in the 20 studies, including 1623 subjects in the RMPP group and 3666 subjects in the NRMPP group. All the 20 studies were evaluated as high quality. All researches were conducted in China, and the earliest research was conducted in 2014, while the latest research in 2021. The baseline characteristics of the included studies are shown in Table 1. The NOS scores of all included studies ranged from 5 to 8, suggesting that all included studies were of high quality

### 3.2. Predictive Factors

#### 3.2.1. CRP

**Figure 2:**
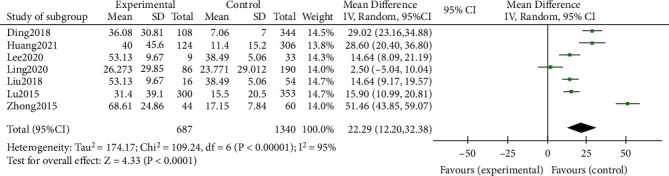
Estimated MD summary for CRP.

A total of 7 articles were merged to analyze CRP, and the heterogeneity test after merging was statistically significant (*I*^2^ = 95%), indicating that the included articles were not homogeneous. So the random-effects model was used. And it was found that the CRP level in the RMPP group was significantly higher than that in the NRMPP group, and the difference was statistically significant (MD (95% CI): 22.29 (12.20, 32.38), *P* < 0.001) (Figure 2).

#### 3.2.2. LDH

**Figure 3:**
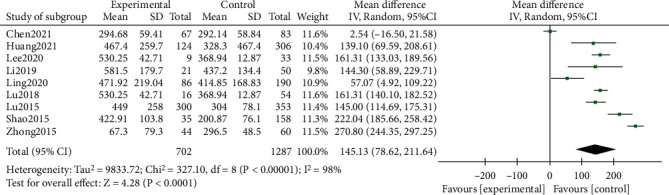
Estimated MD summary for LDH.

A total of 9 studies were merged and analyzed for LDH, and the heterogeneity test after merging was statistically significant (*I*^2^ = 98%). It indicated that the included studies were not homogeneous, so the random effects model was used. And it turned out that the LDH level in the RMPP group was significantly higher than that in the NRMPP group, and the difference was statistically significant (MD (95% CI): 145.13 (78.62, 211.64), *P* < 0.001) (Figure 3).

#### 3.2.3. ESR

**Figure 4:**
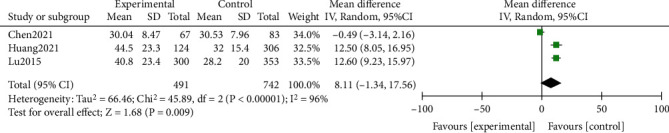
Estimated MD summary for ESR.

Three articles were combined to analyze ESR, and the heterogeneity test after merging was statistically significant (*I*^2^ = 96%), revealing that the included articles are not homogeneous. Therefore, the random effects model was used. The results showed that there was no significant difference in ESR between the RMPP group and the NRMPP group (MD (95% CI): 8.11 (-1.34, 17.56), *P* = 0.09) (Figure 4).

#### 3.2.4. Neutrophils (%)

**Figure 5:**
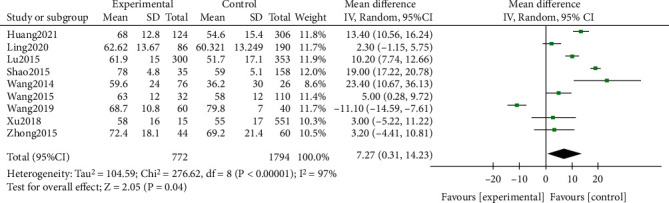
Estimated MD summary for neutrophils (%).

A total of 9 articles were merged and analyzed for neutrophils (%), and the heterogeneity test after merging was statistically significant (*I*^2^ = 97%), indicating that the included articles had poor homogeneity. So the random effects model was used, and the results showed that the neutrophil (%) of the RMPP group was higher than that of the NRMPP group and that the difference was statistically significant (MD (95% CI): 7.27 (0.31, 14.23), *P* = 0.04) (Figure 5).

#### 3.2.5. Lymphocytes (%)

**Figure 6:**
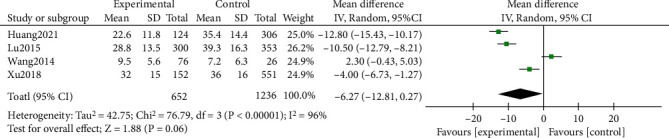
Estimated MD summary for lymphocytes (%).

A total of 4 studies were combined and analyzed for lymphocytes (%), and the heterogeneity test after merging was statistically significant (*I*^2^ = 96%). Therefore, the included papers were not homogeneous, and the random effects model was used. The results showed that there was no significant difference between lymphocytes (%) of the RMPP group and the NRMPP group (MD (95% CI): -6.27 (-12.81, 0.27), *P* = 0.06) (Figure 6).

#### 3.2.6. D-Dimer

**Figure 7:**
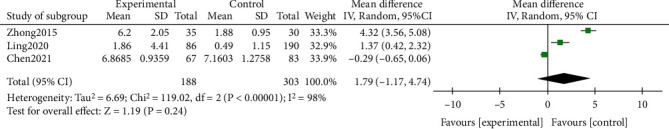
Estimated SMD summary for D-dimer.

A total of 3 articles were merged to analyze D-dimer, and the heterogeneity test after merging was statistically significant (*I*^2^ = 98%), indicating that the included documents have poor homogeneity. So the random effects model was used. It was found that the *D-dimer* in the RMPP group was higher than that in the NRMPP group, and the difference was statistically significant (MD (95% CI): 1.79 (-1.17, 4.74), *P* = 0.24) (Figure 7).

#### 3.2.7. Lung Consolidation

**Figure 8:**
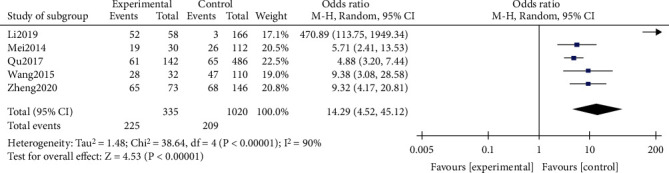
Estimated OR summary for lung consolidation.

A total of 5 studies were combined to analyze lung consolidation, and the heterogeneity test after combination was statistically significant (*I*^2^ = 90%), indicating that the included literature had poor homogeneity. Therefore, a random effects model was used. The results showed that the risk of lung consolidation in the RMPP group was higher than that in the NRMPP group, and the difference was statistically significant (OR (95% CI): 14.29 (4.52, 45.12), *P* < 0.001) (Figure 8).

### 3.3. Sensitivity Analysis

We conducted a sensitivity analysis on each indicator separately to evaluate the reliability of the combined results of each indicator. When each included study was excluded in turn, the results of the combined outcome indicators did not change significantly (see Additional file 1, Figure S1-S7). Therefore, the sensitivity analysis proved the reliability of this meta-analysis.

### 3.4. Publication Bias

Begg's diagram and Egger's diagram were adopted to assess potential publication bias. Due to the limited inclusion of CRP, ESR, D-dimer, lymphocytes (%), and lung consolidation, the results of the publication bias test would also be unreliable due to selection bias, so only LDH and neutrophils (%) were tested. No publication bias was found in LDH (Begg's *P* = 0.548, Egger's *P* = 0.422) and neutrophils (%) (Begg's *P* = 0.754, Egger's *P* = 0.239) (see Additional file 1, Figure S8).

## 4. Discussion

Mycoplasma pneumonia, a pathogen between viruses and bacteria, is a common microscopic organism that causes lower respiratory tract infections in humans. Up to 40% or more of children's community-acquired pneumonia are caused by MP infection. [3, 28] which has caused great clinical attention. Some children with Mycoplasma pneumoniae pneumonia were treated with normal macrolide antibiotics, and their clinical symptoms and imaging manifestations were still worse, indicating RMPP. The study found that the fever time of RMPP was long, and the disease progressed quickly. Large area of lung involvement often occurred in a short period of time, and it was easy to be complicated with pleural effusion and atelectasis. The disease course was prolonged, and the treatment was difficult. Some of these cases can be developed into the acute respiratory distress syndrome, necrotizing pneumonia, or occlusive bronchiolitis severe pneumonia or accompanied by severe pulmonary complications [29].

This study analysis indicators include more inflammatory factors and other features, including CRP, LDH, ESR, *D-dimer*, neutrophils (%), lymphocytes (%), and lung consolidation. A total of 20 studies including 5289 patients were included in the study. And the results showed that the CRP of the RMPP group (MD (95% CI): 22.29 (12.20, 32.38), *P* < 0.001), LDH (MD (95% CI): 145.13 (78.62, 211.64), *P* < 0.001) (Figure 3), neutrophils (%) (MD (95% CI): 7.27 (0.31, 14.23), *P* = 0.04), and D-dimer (MD (95% CI): 1.79 (-1.17, 4.74), *P* = 0.24) was higher than that of the NRMPP group; the risk of lung consolidation in the RMPP group (OR (95% CI): 14.29 (4.52, 45.12), *P* < 0.001) was higher than that in the NRMPP group, and there was no difference in ESR (MD (95% CI): 8.11 (-1.34, 17.56), *P* = 0.09); lymphocytes (%) (MD (95% CI): -6.27 (-12.81, 0.27), *P* = 0.06) were closely related to RMPP. The final prediction results also revealed that these indicators could also have a better predictive value under a certain cutoff value.

CRP, LDH, and ESR are nonspecific inflammatory factors in the body, and previous studies have different conclusions in the study of the correlation with RMPP. CRP is an acute protein. When the body has tissue damage caused by inflammation and infection, CRP will increase, which is an important indicator for the diagnosis of childhood pneumonia. As a cytoplasmic enzyme, LDH exists in various important organs. When the cell is dissolved or the cell membrane is destroyed, LDH can be released outside the cell, causing an increase in LDH in the serum. After the combined analysis, we found that the increase in CRP and LDH was highly correlated with RMPP. Previous studies have shown that ESR is a predictive factor for the development of RMPP. However, in our meta-analysis, it was found that the ESR levels of RMPP and NRMPP were not different after the merger of the three studies [9, 12, 27], which is contrary to the previous conclusions. This may be related to the small number of studies, and more researches are needed to prove this point in the future. We also found that neutrophils (%) in children with RMPP were higher than those in the NRMPP group, which may be related to bacterial infection. After Mycoplasma pneumoniae adheres to the surface of epithelial cells, it will continue to damage the ciliated columnar epithelium, resulting in a decrease in the number of cilia. The abnormal structure of the cilia can also cause damage to the clearance of the mucociliary system. As a result, some pathogenic bacteria will continue to multiply, causing mixed infections in children. The imaging changes in children with RMPP are more obvious; the most common is unilateral or bilateral large-scale consolidation. Through a pooled analysis, we found that the risk of lung consolidation in children with RMPP was 14.286 times that of children with NRMPP, which seems helpful for the early diagnosis of RMPP. However, we also suspect that prolonged inflammation after infection might be a possible reason for lung consolidation and RMPP. Therefore, the conclusion that lung consolidation is a risk factor for RMPP is open to question. Further research is needed to confirm in the future.

In addition, we also describe the prediction of RMPP by different indicators. There is a previous meta-analysis for the prediction of community-acquired pneumonia [30] which mainly studied the efficacy of CRP alone or in combination in diagnosing community-acquired pneumonia under different cutoff values. We cannot carry out the same quantitative data analysis as this study. Six studies [12, 17, 18] reported the data of CRP to predict RMPP. But one study [18] only reported AUC, so we cannot know its cutoff value, sensitivity, and specificity. However, according to the existing data, when the cutoff value was 51 mg/L, the maximum AUC was 0.917, and the prediction effect was the best. In this case, the sensitivity was 97.1%, and the specificity was 96.8%. When the cutoff value was 17.5 mg/L, CRP had the worst predictive effect of RMPP. On this condition, the AUC was 0.35, while the sensitivity was 73.3%, and the specificity was 55.0%. Seven studies [10, 12, 14, 17] reported the data of LDH for RMPP prediction. When the cutoff value was 314.5 IU/L, LDH had the worst predictive effect of RMPP, with an AUC of 0.147, the sensitivity of 79.7%, and the specificity of 65%. When the cutoff value was 353 IU/L, LDH had the best effect in predicting RMPP, with an AUC of 0.900, the sensitivity of 85.7%, and the specificity of 92.4%.Only one study [10] analyzed the effect of ESR in predicting RMPP. At this time, the cutoff was 16.5 IU/L. Under this cutoff value, the AUC was 0.718, while the sensitivity and specificity were 62% and 66.4%, respectively, indicating that the prediction effect was not good. There are two studies on the effect of neutrophils (%) in predicting RMPP. Compared with the cutoff of 68.6, the cutoff of 71 had a better predictive effect; as the AUC was 0.91, the sensitivity was 88.6%, and the specificity was 93.7% (see Additional file 1, Table S1).

According to the existing data, we can only know that the best cutoff point of LDH is 353 when the AUC is the largest and the diagnosis effect is the best [15]. The best cutoff value of CRP has the best diagnostic effect at 51 [15]. It is also unable to determine the diagnostic efficacy of ESR because of only one study of data [12]. In the future, more clinical studies are needed to study the diagnostic efficacy under different cutoff values.

The advantage of our meta-analysis lies in the systematic analysis of predictive factors and predictive analysis that has never been done before. And two independent researchers were responsible for data extraction and quality assessment to reduce errors. But there are still some limitations. After combined analysis, it was found that the heterogeneity between studies was large, and no suitable subgroup was found to explore the source of heterogeneity. However, we hypothesized that the time of macrolide administration between NRMPPS might be one of the reasons for heterogeneity, and differences in living and treatment environments might also lead to heterogeneity between studies. All the included studies were conducted in China, which may lead to the inapplicability of the results to other ethnic groups. We speculate that China's large population base may make it easy to obtain people who meet the inclusion criteria.

In addition, the overall number of documents is not large. We originally wanted to focus on the prediction research of RMPP, but the existing articles and data are not enough for us to find a predictive diagnosis, and the current data is all from China. For the best cutoff value, more prospective studies are needed in the future to study the diagnostic efficacy of different cutoff values. In addition to sensitivity, we also wanted to conduct an analysis of publication bias. But since there are fewer than 10 articles for each indicator, even if it is done, the results will still not be of great significance in practice, so it was not carried out.

## 5. Conclusions

According to current studies, CRP, LDH, neutrophil (%) elevation, and lung consolidation are predictive factors of RMPP. By monitoring these factors, early prevention can be carried out for patients with RMPP, and timely measures can be taken to improve the prognosis of those who have already occurred. However, due to the limitations of this study, the correlation between the results and the remaining risk factors needs to be verified by more rigorous clinical trials. However, due to the limitations of the studies included in this paper, it cannot be stated which indicator is the most accurate indicator for predicting the severity of RMPP. Therefore, in future studies, more high-quality, large-sample diagnostic studies or combined predictive studies are needed to confirm our conclusions.

## Figures and Tables

**Table 1:** Baseline characteristics.

Author	Year	Country	RMPP			NRMPP			Quality assessment
			Total	M/F	Age, years	Total	M/F	Age, years	
Wang et al. [11]	2014	China	76	42/34	5.55 ± 3.25	26	14/12	4.03 ± 2.95	7
Mei et al. [10]	2014	China	30	18/12	6.0 (5.0 ~ 9.0)	112	61/51	5.0 (3.0 ~ 6.0)	6
Lu et al. [12]	2015	China	300	—	—	353	—	—	6
Xiao et al. [13]	2015	China	44	20/2	3.3 ~ 13.6	60	28/32	2.1 ~ 14.3	7
Wang et al. [14]	2015	China	32	13/19	6.9 ± 2.8	110	60/50	5.7 ± 2.8	7
Shao et al. [15]	2015	China	35	19/16	—	158	68/90	—	6
Zhang et al. [3]	2016	China	145	70/75	5.9 (3.8 ~ 8.0)	489	280/209	3.4 (1.9 ~ 6.3)	6
Zhai et al. [16]	2017	China	142	70/72	6.84 ± 2.51	286	282/204	4.63 ± 1.94	7
Ding et al. [17]	2018	China	108	54/54	5.21 ± 2.92	344	198/146	2.98 ± 2.81	6
Liu et al. [18]	2018	China	16	6/10	5.84 ± 0.82	54	27/27	7.17 ± 0.60	5
Xu and Shu [19]	2018	China	152	272/279	5.1 ± 2.9	551	70/82	4.8 ± 2.9	6
Li et al. [20]	2019	China	21	11/10	5.38 ± 2.3	50	18/32	5.38 ± 2.83	6
Wang et al. [21]	2019	China	60	24/36	6.7 (4.7 ~ 7.5)	40	17/23	5.5 (4.5 ~ 8.0)	8
Li et al. [22]	2019	China	58	36/22	6.3 ± 2.7	166	120/46	3.4 ± 1.3	7
Lee et al. [23]	2020	China	9	3/5	5.84 ± 0.82	33	—	7.17 ± 0.60	6
Ling et al. [24]	2020	China	86	38/48	6 (4-8)	190	112/78	6 (4-7)	6
Zhao et al. [25]	2020	China	45	28/17	9.3 ± 2.7	109	62/47	8.8 ± 2.8	6
Zheng et al. [26]	2020	China	73	38/35	6.5 ± 2.5	146	79/67	6.4 ± 2.8	7
Chen et al. [9]	2021	China	67	39/28	4.73 ± 2.65	83	52/31	4.31 ± 2.06	8
Huang et al. [27]	2021	China	124	61/63	5.7 ± 2.7	306	176/130	4.3 ± 2.5	7

## Data Availability

The datasets used and/or analyzed during the current study are available from the corresponding author on reasonable request.

## Abbreviations

CAP: Community-acquired pneumonia
RMPP: Refractory Mycoplasma pneumoniae pneumonia
WMD: Weighted mean difference
NOS: Newcastle–Ottawa Scale.

## Supplementary Materials

Supplementary Materials Figure S1: sensitivity analysis of CRP. Figure S2: sensitivity analysis of LDH. Figure S3: sensitivity analysis of ESR. Figure S4: sensitivity analysis of neutrophils (%). Figure S5: sensitivity analysis of lymphocytes (%). Figure S6: sensitivity analysis of D-dimer. Figure S7: sensitivity analysis of lung consolidation. Figure S8: publication bias tested by Begg's test and Egger's test. (A) Begg's test for LDH; (B) Egger's test for LDH; (C) Begg's test for neutrophils (%); (D) Egger's test for neutrophils (%). Table S1: predicted RMPP results of various indicators.
