# Supplementary material for: Refractory Mycoplasma Pneumonia in Children: A Systematic Review and Meta-analysis of Laboratory Features and Predictors
Source: J Immunol Res. 2022 Jun 26;2022:9227838. doi: 10.1155/2022/9227838 (PMC9251082; doi:10.1155/2022/9227838)
Supplement: Supplementary Materials — Figure S1: sensitivity analysis of CRP. Figure S2: sensitivity analysis of LDH. Figure S3: sensitivity analysis of ESR. Figure S4: sensitivity analysis of neutrophils (%). Figure S5: sensitivity analysis of lymphocytes (%). Figure S6: sensitivity analysis of D-dimer. Figure S7: sensitivity analysis of lung consolidation. Figure S8: publication bias tested by Begg's test and Egger's test. (A) Begg's test for LDH; (B) Egger's test for LDH; (C) Begg's test for neutrophils (%); (D) Egger's test for neutrophils (%). Table S1: predicted RMPP results of various indicators. [file 9227838.f1.docx]

Pubmed Search strategy:

((((Mycoplasma pneumoniae pneumonia[Title/Abstract]) OR (MPP[Title/Abstract])) OR (refractory Mycoplasma pneumoniae pneumonia[Title/Abstract])) OR (RMPP[Title/Abstract])) AND ((children[Title/Abstract]) OR (child[Title/Abstract]))

Table S1 Predicted RMPP results of various indicators

| Study | Cutoff | Sensitivity | Specificity | AUC |
| --- | --- | --- | --- | --- |
| CRP(mg/L) |  |  |  |  |
| Zhang,2016[3] | 16.5 | 74.7 | 77.2 | 0.817 |
| Zhao,2020[25] | 12.1 | 73.3 | 55.0 | 0.655 |
| Chen,2021[9] |  |  |  | 0.862 |
| Shao,2015[15] | 51 | 97.1 | 96.8 | 0.917 |
| Li,2019[20] | 17.5 | 50.0 | 48.0 | 0.35 |
| Zhai,2017[18] | 15.3 | 77.6 | 79.5 | 0.821 |
| LDH(IU/L) |  |  |  |  |
| Lu,2015[12] | 379 | 48 | 85.5 | 0.718 |
| Zhang,2016[3] | 417 | 79.7 | 65 | 0.772 |
| Liu,2018[18] | 408 | 75 | 72.2 | 0.812 |
| Zhao,2020[25] | 436.5 | 77.8 | 62.4 | 0.775 |
| Shao,2015[15] | 353 | 85.7 | 92.4 | 0.9 |
| Li,2019[20] | 314.5 | 44.0 | 35.0 | 0.147 |
| Zhai,2017[18] | 402 | 81.5 | 66.3 | 0.783 |
| ESR(IU/L) |  |  |  |  |
| Lu,2015[12] | 32.5 | 62 | 66.4 | 0.718 |
| Neutrophils(%) |  |  |  |  |
| Shao,2015[15] | 71.0 | 88.6 | 93.7 | 0.91 |
| Zhang,2016[3] | 68.6 | 68.4 | 81.1 | 0.803 |


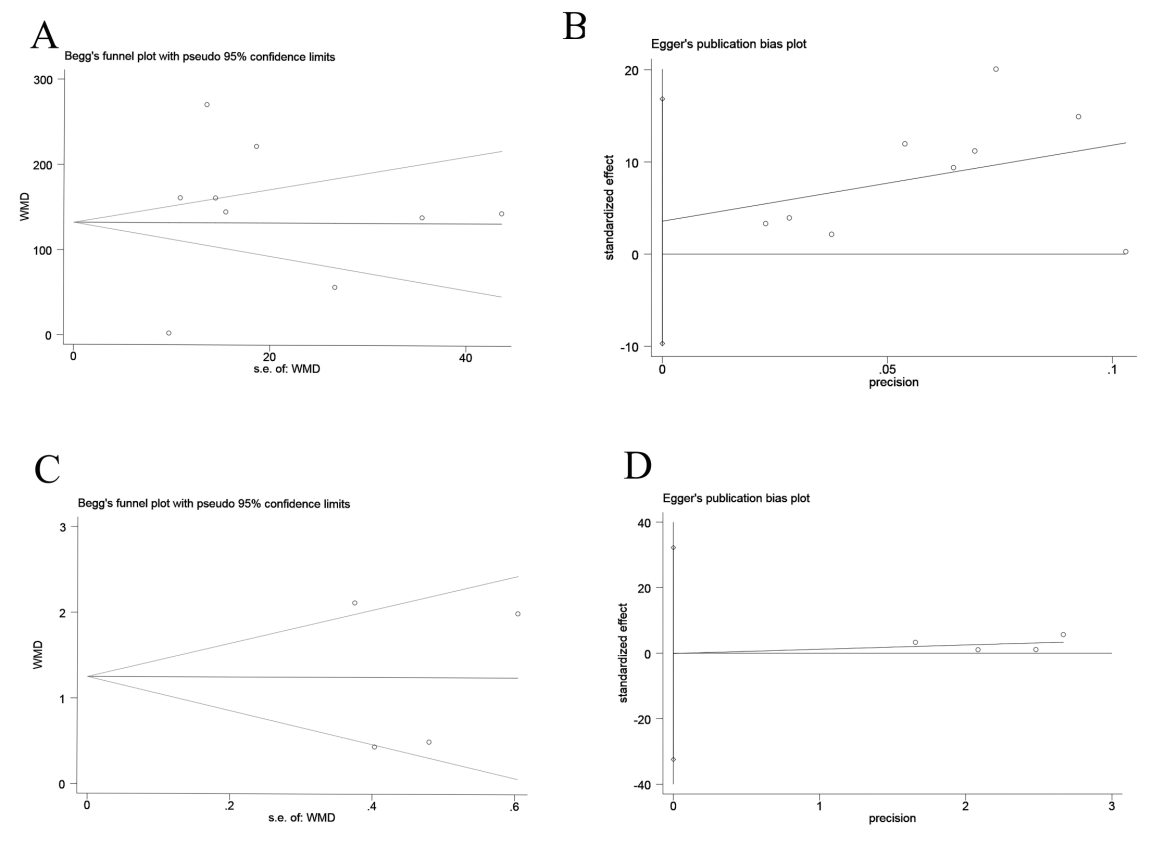


Figure S1 Publication bias tested by Begg’s test and Egger’s test. (A) Begg’s test for LDH; (B) Egger’s test for LDH; (C) Begg’s test for Neutrophils (%); (D) Egger’s test for Neutrophils (%)

Sensitivity analysis:

FigureS2: Sensitivity analysis of CRP

FigureS3: Sensitivity analysis of LDH

FigureS4: Sensitivity analysis of ESR

FigureS5: Sensitivity analysis of Neutrophils (%)

FigureS6: Sensitivity analysis of Lymphocytes (%)

FigureS7: Sensitivity analysis of D-Dimer

FigureS8: Sensitivity analysis of Lung consolidation


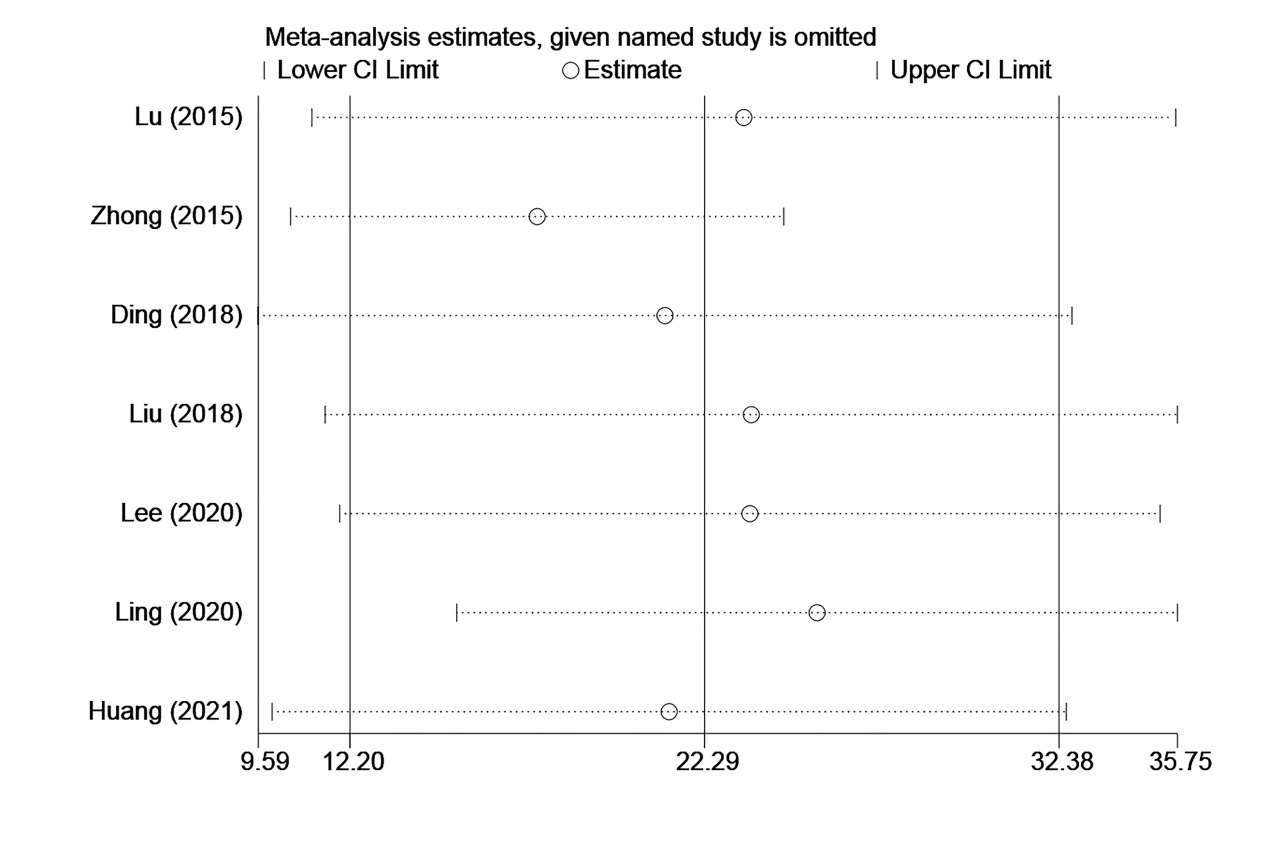


Figure S2 Sensitivity analysis of CRP


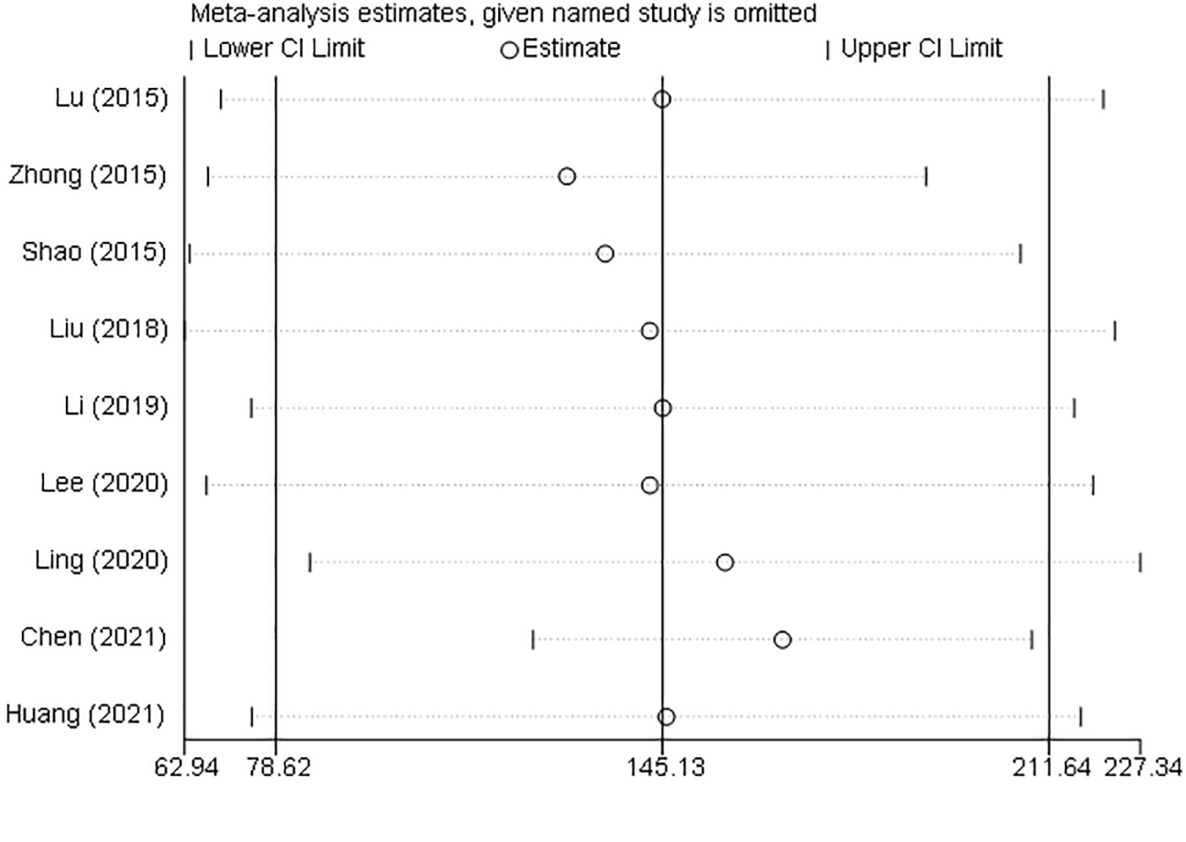


Figure S3 Sensitivity analysis of LDH


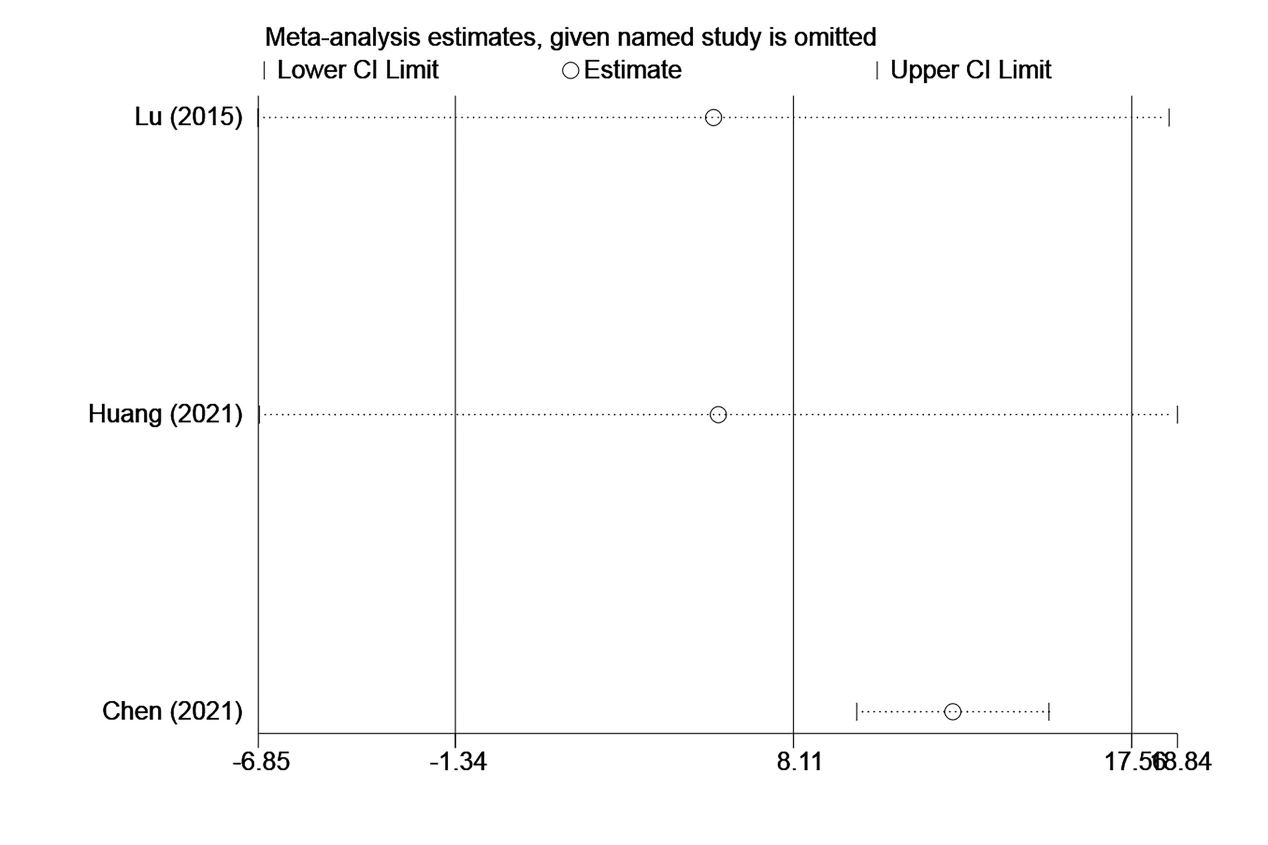


Figure S4 Sensitivity analysis of ESR


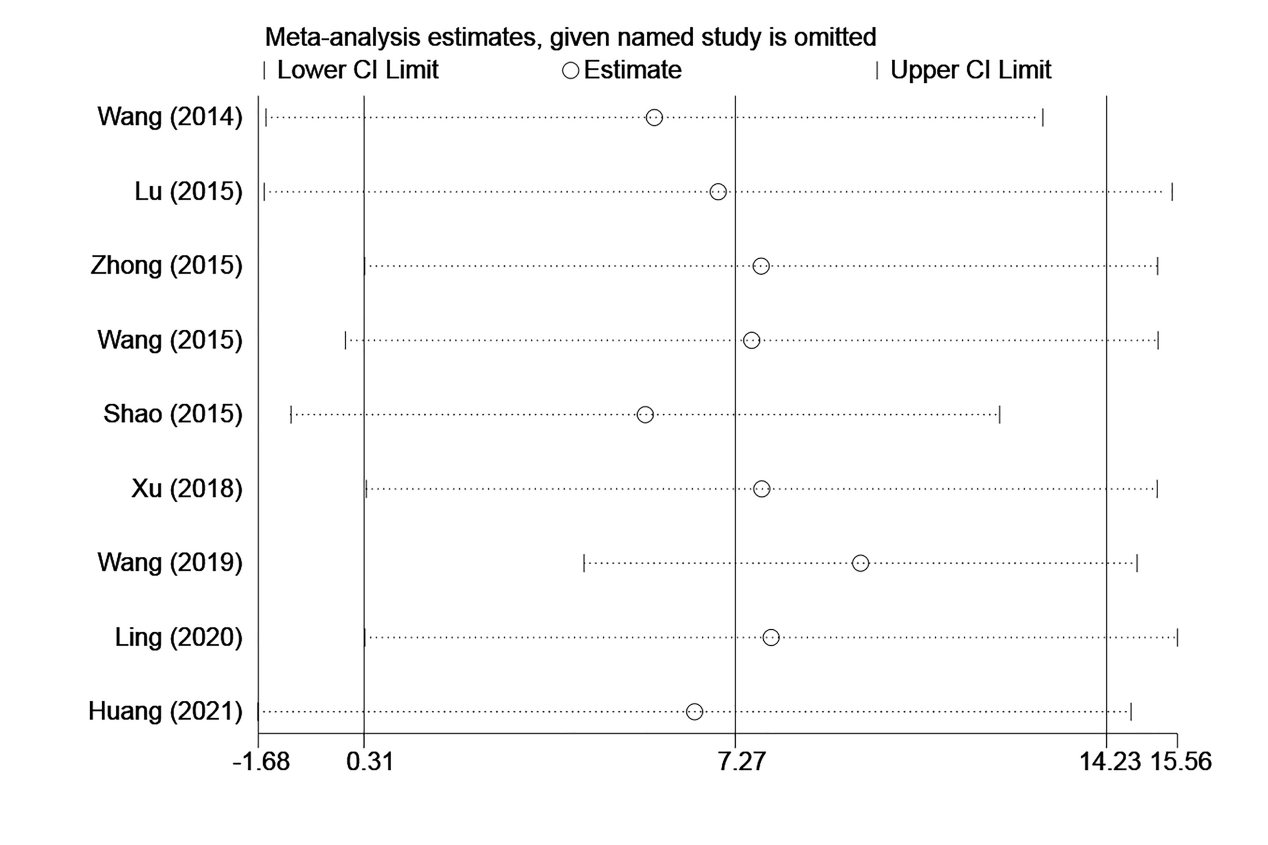


Figure S5 Sensitivity analysis of Neutrophils (%)


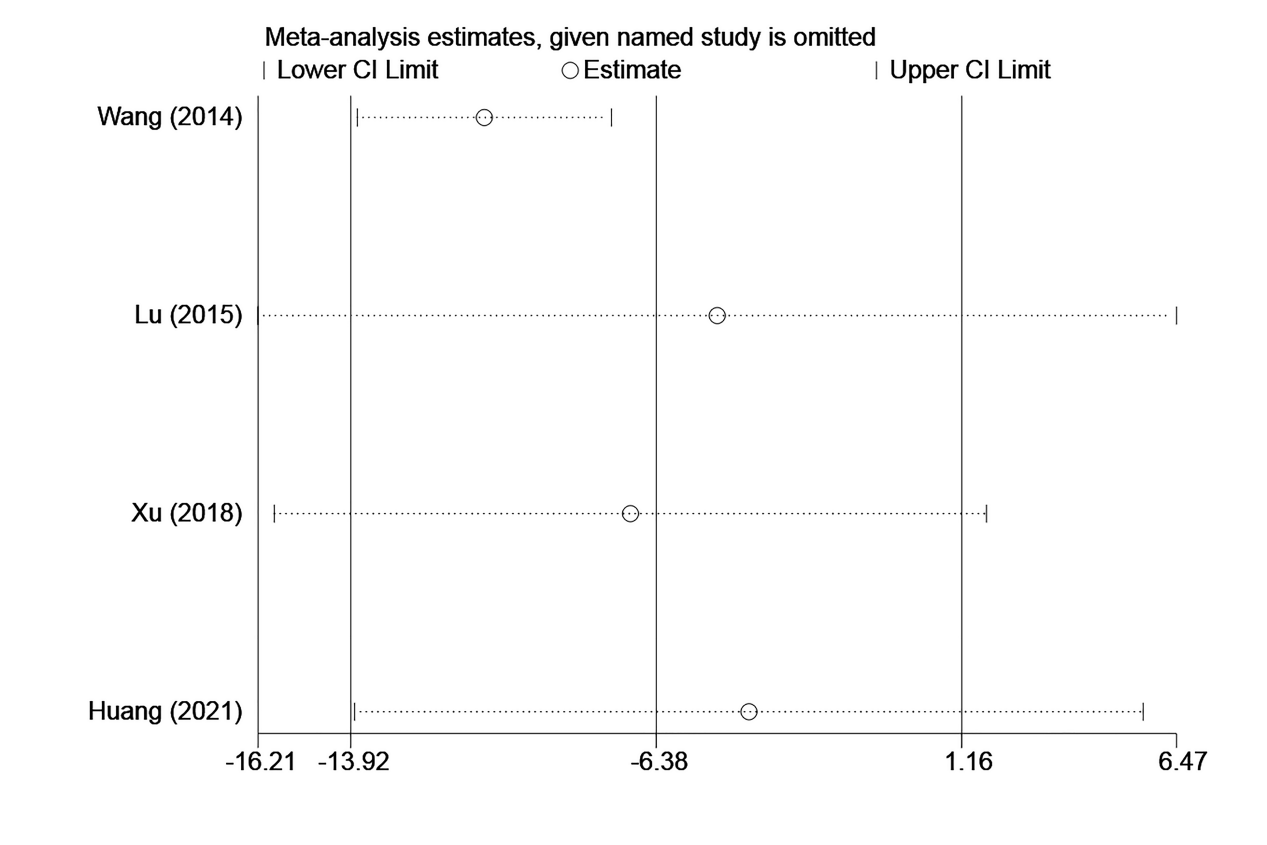


Figure S6 Sensitivity analysis of Lymphocytes (%)


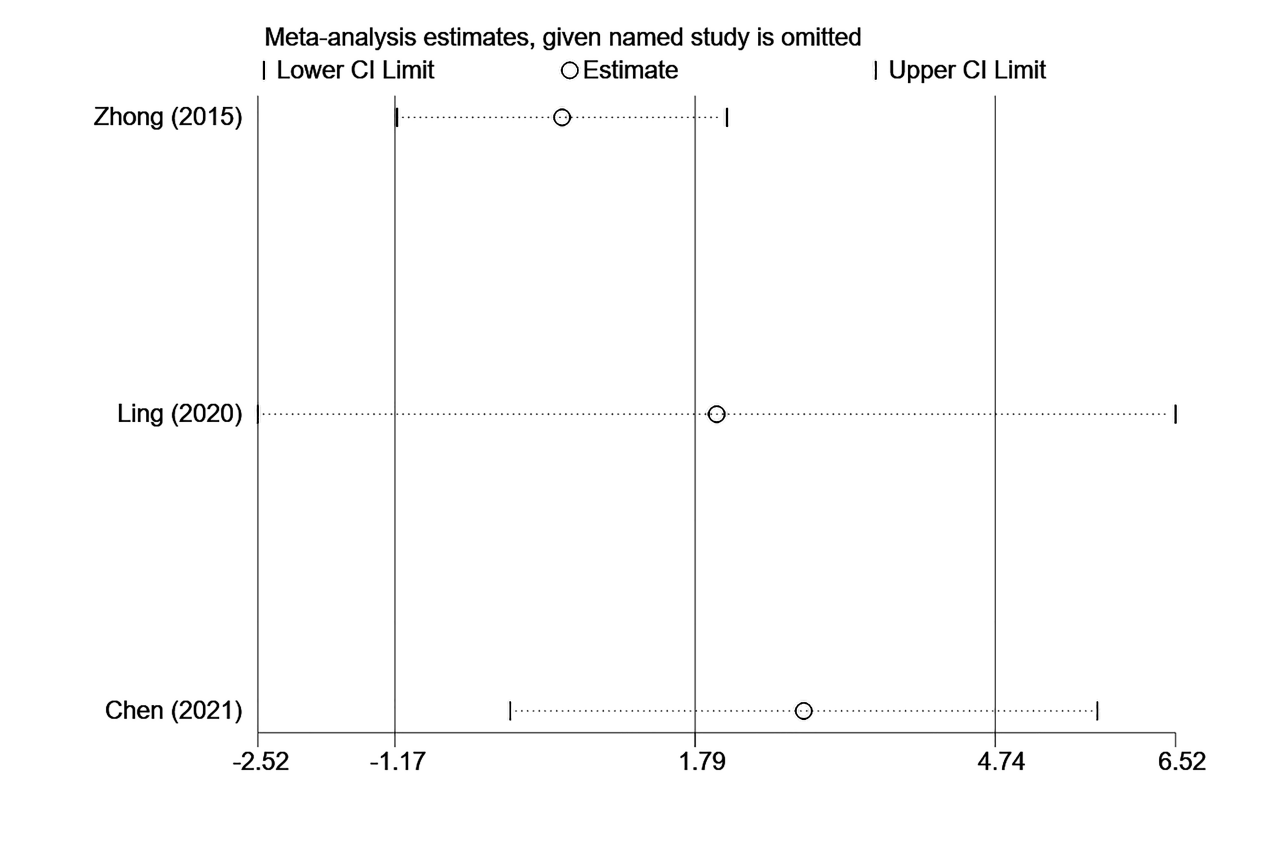


Figure S7 Sensitivity analysis of D-Dimer


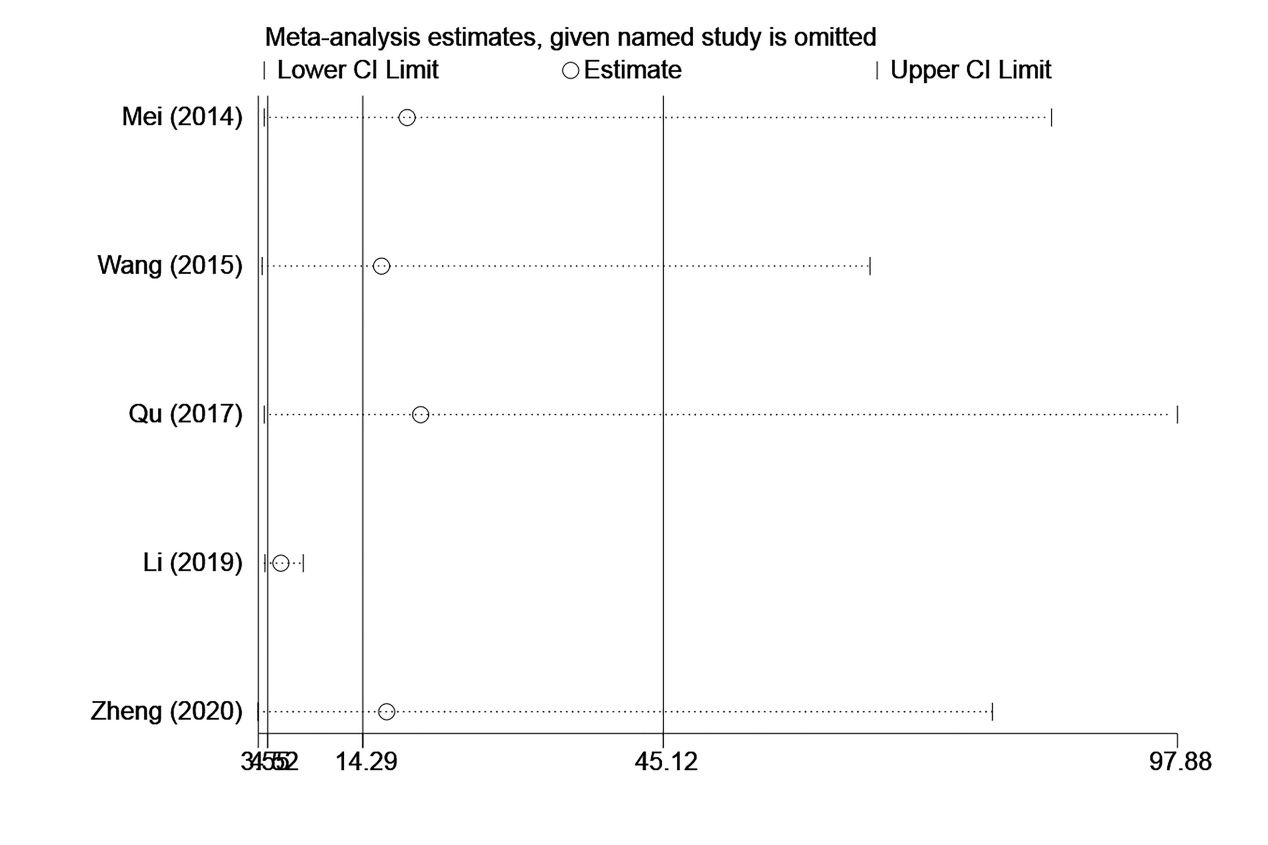


Figure S8 Sensitivity analysis of Lung consolidation
